# Analysis and comparison of the trends in burden of atrial fibrillation/atrial flutter in China and worldwide from 1990 to 2021 and predictions to 2036 of China

**DOI:** 10.3389/fcvm.2025.1540750

**Published:** 2025-05-30

**Authors:** Zhilin Wang, Zhenghua Shi, Jiayi Shen, Susu Zhang, Yunteng Fang, Wuming Hu, Lingchun Lv

**Affiliations:** ^1^ Department of Cardiology, Zhejiang University Lishui Hospital, Hangzhou, China; ^2^ Department of Cardiology, Lishui Central Hospital and the Fifth Affiliated Hospital of Wenzhou Medical University, Lishui, China

**Keywords:** atrial fibrillation/atrial flutter (AF/AFL), global burden of disease (GBD), socio-demography index (SDI), inequality, prediction, disability-adjusted life years (DALYs), joinpoint regression, age-period-cohort (APC) analysis

## Abstract

**Background:**

It is essential to analyze the burden, trends, and inequalities of atrial fibrillation/flutter (AF/AFL) in China and to predict future trends, with the aim of raising awareness about risk factors and exploring strategies to control the significant disease burden.

**Methods:**

Data pertaining to AF/AFL were extracted from the comprehensive dataset of the Global Burden of Disease, Injuries, and Risk Factors Study 2021 (GBD 2021). Furthermore, we analyzed the epidemiological characteristics of AF/AFL and compared them to global prevalence trends, employing joinpoint regression, decomposition, age-period-cohort (apc), and cross-country inequality analysis methods. Concurrently, we utilized a Bayesian age-period-cohort (BAPC) analysis to forecast the age-standardized incidence rate (ASIR) of AF/AFL in China over the subsequent 15 years.

**Results:**

Globally, in 2021, there were 52.55 million [95% uncertainty interval (UI): 43.14 to 64.96] prevalent cases, 4.48 million (95% UI: 3.61 to 5.71) incident cases, 0.34 million (95% UI: 0.28 to 0.37) deaths, and 8.36 million (95% UI: 6.97 to 10.13) DALYs. In China, during the same year, there were 10.78 million (95% UI: 8.53 to 14.01) prevalent cases, 0.92 million (95% UI: 0.71 to 1.20) incident cases, 0.06 million (95% UI: 0.05 to 0.08) deaths, and 1.65 million (95% UI: 1.30 to 2.06) DALYs. The average annual percentage change (AAPC) in age-standardized incidence and mortality rates for AF/AFL were −0.02 (95% CI: −0.05, 0) and 0.11 (95% CI: 0.03, 0.18) globally, and 0.16 (95% CI: 0.05, 0.26) and −0.45 (95% CI: −0.78, −0.12) in China, respectively. Decomposition analysis revealed epidemiological shifts drive incidence rise, aging affects mortality. The Slope Index of Inequality (SII) 2021 was −35.04, and the Concentration Index (CI) was −0.09. The BAPC results indicated that the ASIR for males and females is expected to rise over the next 15 years.

**Conclusion:**

The burden of AF/AFL continues to increase annually. Countries with medium to low Socio-Demographic Index (SDI) have a heavy disease burden. In recent years, the burden in Chinese females has begun to exceed that of males. Without effective measures, the ASIR of AF/AFL is projected to exhibit a continued upward trajectory.

## Introduction

Cardiovascular disease (CVD) continues to be a primary contributor to premature mortality and escalating healthcare expenditures (1). Although ischemic heart disease has always been the leading cause of global disease burden in CVD and ranks first in the age-standardized mortality rate from 1990 to 2021 (2), in recent years, the disease burden associated with other CVDs has also been gradually increasing globally, including in China. AF/AFL are among the most prevalent forms of arrhythmia, potentially resulting in numerous complications, such as stroke and heart failure (HF), and possessing a multitude of risk factors that can precipitate these conditions. Furthermore, HF is identified as the leading cause of mortality in the patients presenting with clinical AF (3). The global incidence and prevalence of AF are escalating steadily. According to the statistics provided by the Framingham Heart Study (FHS), the prevalence of AF has increased threefold over the past 50 years (4). The Global Burden of Disease initiative has assessed the global incidence of AF to be roughly 60 million individuals in the year 2019 (5). In the United States, it is estimated that between 3 and 6 million individuals currently suffer from AF, with projections suggesting that this number could increase to between 6 and 16 million by 2050 (6). In Europe, the prevalence of atrial AF within the demographic aged 55 and above was estimated at 9 million in 2010 (7), with projections indicating a rise to 14 million by the year 2060 (8). It was projected that by the year 2050, the diagnosis of AF will affect a minimum of 72 million individuals across Asia, with an estimated 3 million cases leading to strokes associated with AF (9). Certainly, a host of people have asymptomatic atrial fibrillation, so the global AF/AFL burden are underestimated (10). The incidence and mortality rates of AF/AFL rise with advancing age (4). Furthermore, previous research has shown that risk factors encompass sex, smoking, alcohol consumption, body mass index, hypertension, heart failure, obstructive sleep apnea, left ventricular hypertrophy, and myocardial infarction (11, 12). Cheng et al. (13) recently published a study demonstrating that elevated systolic blood pressure is the primary risk factor for the growing global burden of AF/AFL, with high BMI being a close second. There is also evidence indicating that psycho social and lifestyle factors play significant roles as modulating influences on the incidence of AF, particularly among younger individuals (14).

China, as the most populous country globally, has experienced significant changes in its economic development and social context (15). Concurrently, the relevant risk factors are also in effect, resulting in a distinctive trend of disease burden for AF/AFL within China. Consequently, within China, performing corresponding analysis utilizing the GBD 2021 database to investigate the distribution characteristics among various populations, and forecasting future trends, can offer valuable epidemiological data for the development of public health policies and to enhance awareness for disease prevention. The principal aim of this study is to delineate the trends in the prevalence, incidence, mortality, and DALYs of AF/AFL, forecast the changes to 2036, by which time China will have basically achieved socialist modernization. Furthermore, performing joinpoint regression analysis and cross-country inequality analysis on the burden of AF/AFL individually for China and on a international context provides supplementary insights to the previously conducted global disease burden assessment of AF/AFL.

## Materials and methods

### Study population and data collection

The Institute for Health Metrics and Evaluation (IHME) has released a comprehensive study entitled The Global Burden of Disease, Injuries, and Risk Factors 2021 (GBD 2021), marking the most extensive and rigorous endeavor to assess the global impact of diseases and their associated risk factors (2). The latest installment of the Global Burden of Disease (GBD) series, GBD 2021, has broadened its evaluative scope to include 371 distinct diseases and injuries, along with 88 risk factors, across the same geographic distribution from 1990 to 2021 (16). This methodological diversity is crucial for capturing the comprehensive impact of health conditions worldwide. A thorough and detailed exposition of the processes employed for burden estimation, including the calculation of key metrics such as incidence, prevalence, mortality, years of life lost (YLLs), years lived with disability (YLDs), and disability-adjusted life-years (DALYs) within the GBD 2021, has been meticulously documented in peer-reviewed publications. These detailed methodologies underscore the rigorous scientific approach of the GBD 2021 study, ensuring the accuracy and reliability of its findings, which are vital for informing global health policy and research agendas (16). The study population comprised people with AF/AFL of all ages and age-standardized. The core data underpinning our analysis were sourced from the Global Burden of Diseases, Injuries, and Risk Factors Study (GBD) 2021, leveraging the interactive capabilities of the Global Health Data Exchange's online query tool (https://ghdx.healthdata.org/gbd-results-tool) (1). Based on the Global Burden of Disease Study 2021, we have meticulously extracted comprehensive data pertaining to AF/AFL across various age groups, encompassing details such as geographical location, age distribution, and specific prevalence rates. Additionally, we have included insights into mortality figures, along with the counts and rates of DALYs [with corresponding 95% uncertainty intervals (UI)s], offering a holistic understanding of the impact of these conditions. The comprehensive details and methodologies employing the GBD 2021 study have been meticulously documented and published in prior scholarly works (1, 17). The Institutional Review Board at the University of Washington has conducted a meticulous review and subsequently granted an exemption for the requirement of informed consent for the use of de-identified, consolidated data within the GBD 2021 study. Adherence to all relevant guidelines and regulations was rigorously observed throughout the methodology implementation.

### Definition

In our study, instances of Atrial Fibrillation and Flutter (AF/AFL) were meticulously ascertained by employing the standardized International Classification of Diseases and Injuries (ICD) coding system, ensuring a rigorous and systematic approach to case identification. Specifically, we utilized ICD-9 codes ranging from 427.3 to 427.32 and ICD-10 codes from I48 to I48.92 to categorize cardiovascular diseases as AF/AFL (18). In accordance with prior GBD studies (5, 13, 17, 19) electrocardiogram was used to diagnose AF/AFL.

Disability-adjusted life years (DALYs): DALYs is a measure used to quantify health loss. It combines the impact of death and disability due to diseases or injuries. DALYs is a composite indicator that combines years of life lost due to premature mortality (YLLs) with years lived with disability (YLDs). DALYs is a tool for assessing the burden of disease, helping public health experts and policymakers understand which diseases and injuries have the greatest impact on population health (20).

Age-standardized DALYs: It is an epidemiological indicator used to measure and compare health loss across different populations or at different time points. The purpose of age standardization is to adjust to the impact of differences in age structure on DALYs, making comparisons fairer and more accurate (16).

The Socio-Demographic Index (SDI): The SDI serves as a comprehensive indicator, utilized to evaluate the extent of social development within a region or country. It is closely related to health outcomes and is often used in Global Burden of Disease (GBD) studies. The SDI takes into account several key socioeconomic factors, including per capita income, level of education, and fertility rate, especially the fertility rate of women under 25, which is related to the status of women. A higher fertility rate may indicate that society does not place enough emphasis on the status of women (16). SDI is also used to analyze changes in healthy life expectancy, and in many cases, it can explain most of the variation in healthy life expectancy. For example, a higher SDI in a country generally means that its people can expect a longer healthy life span. Moreover, the increase in SDI is often associated with overall societal progress, such as economic development, increased educational opportunities, and the elevation of women's status. The countries and territories were classified into five distinct regions, each categorized based on their SDI: high, high-middle, middle, low-middle, and low (21). SDI values, ranging on a continuum from 0 to 1, are indicative of a nation's level of development, with higher indices denoting greater developmental status (22).

### Joinpoint regression analysis

In our study, we specifically used the joinpoint regression model, which consists of a series of linear statistical models, to assess the temporal trends in the disease burden attributable to AF. This model's computational methodology involves estimating various patterns of disease incidence rates using the least squares method, thereby effectively avoiding the inherent subjectivity of traditional trend analyses that rely solely on linear trends. The model identifies the pivotal point of trend deviation by quantifying the aggregate squared discrepancies between the anticipated and observed outcomes. We utilized Joinpoint (23) to create this model. Additionally, we calculated the average annual percentage change (AAPC) and annual percentage change (APC), focusing on statistically significant fluctuations across different segments by comparing the AAPC to 0. Joinpoint regression analysis, by identifying multiple inflection points within a time series, segments the data into distinct trend phases and fits models separately, thereby precisely capturing complex evolutionary processes. This approach surpasses traditional methods that rely on a single trend assumption. Specifically, Joinpoint regression provides the slope of each phase and the confidence intervals of inflection points, quantifying the direction, rate, and significance of trend changes. This offers a refined statistical basis for analyzing temporal heterogeneity in indicators such as Age-Standardized Incidence Rates (ASIR). The strength of this method lies in its flexibility to adapt to the actual patterns of data variation, particularly in scenarios involving complex trends with multiple inflection points. It overcomes the limitations of traditional models that presuppose a specific trend form. Through dynamic segmentation of trend intervals and statistical testing, Joinpoint regression achieves objective identification and robust interpretation of structural changes in non-stationary time series (24). A *p*-value of less than 0.05 was considered statistically significant (25).

### Age-period-cohort analysis

Age-period-cohort (APC) models are a cornerstone in the fields of sociology and epidemiology, leveraging Poisson distributions to adeptly capture and analyze temporal dynamics in incidence or mortality rates. These models dissect time-related trends into distinct yet interconnected components of age, period, and cohort, offering a sophisticated perspective through examining the influence of time on health outcomes. However, the inherent linear relationship between these variables poses challenges in uniquely estimating effects for each age, period, and cohort, potentially leading to issues of non-identifiability (26). Researchers have explored diverse angles to tackle this problem, introducing various solutions like intrinsic estimators (27), penalty function methods (28), and estimation functions (29), yet these approaches still encounter limitations. B. Carstensen offers a comprehensive explanation of an analytical technique for APC models, leveraging the Lexis diagram (30, 31). In this study, we utilized a specific methodology for APC analysis. We queried the Global Burden of Disease (GBD) database to obtain incidence and mortality data, stratified by 5-year age groups, for the period spanning from 1992 to 2021. Additionally, we retrieved population estimates for each year from the same source (https://ghdx.healthdata.org/record/ihme-data/global-population-forecasts-2017-2100). It is noteworthy that GBD combines individuals under 5 and over 95 years into a single category. To facilitate the fitting of the Age-Period-Cohort (APC) model, we categorized the population into age groups ranging from 0 to 4 to 95–100 years, with the age group 0 representing children under five years old, as illustrated in the figures. For each 5-year interval from 1992 to 2021, we calculated the total incidence and mortality counts, as well as the cumulative incidence and mortality rates, across these age groups (32). To fit the APC model, we used the Epi package (version 2.55) within the R statistical environment (version 4.4.1 https://www.r-project.org).

### Decomposition analysis

We performed decomposition analysis to visually explore the significance of three pivotal factors influencing variations in incidence and mortality rates from 1990 to 2021 in China: aging, population dynamics, and epidemiological changes. Epidemiological changes pertain to the underlying shifts in age-specific, morbidity rates, and population-adjusted mortality (33).

### Cross-country inequality analysis

To quantify the disparities in the burden of AF/AFL across various countries, we utilized the Slope Index of Inequality (SII) and Concentration Index (CI), both of which are based on the SDI (34). The SDI-based relative position scale was employed to calculate the SII, which involved regressing the prevalence of AF/AFL across all age populations at the country level against the SDI-related position. This position is determined by the midpoint of the cumulative class range of the population sorted by SDI. Furthermore, the CI for Health Inequality was derived by aligning a Lorenz Concentration Curve with the empirical cumulative relative distribution of the population. This alignment was based on categories defined by SDI and disease prevalence, followed by a numerical integration of the area beneath the curve (35). This methodology provides a robust measure of health inequality, reflecting the disparities in disease burden across different socioeconomic stage.

### Predictive model

To ascertain the progression of ASIR trends for AF/AFL subsequent to the year 2021 in China, a BAPC analysis was executed within the R statistical environment, employing the BAPC, Nordpred, and INLA packages (36, 37). We employed the Nordpred package in R to conduct age-period-cohort (APC) analysis stratified by gender, projecting the number of new cases and deaths from esophageal cancer from 2021 to 2036. This approach accounted for both changing rates and evolving population structures, a methodology that has been extensively validated and recognized in prior research. To facilitate comparison with the projected outcomes, we calculated the absolute number of events under three scenarios based on the rates observed in 2019: a stable rate scenario (baseline reference), an annual 1% reduction scenario (optimistic reference), and an annual 1% increase scenario (pessimistic reference). To assess the robustness of our predictions, we further applied a BAPC model using the integrated nested Laplace approximation (INLA) for sensitivity analysis, implemented through the BAPC and INLA packages in R (38). This analytical process facilitated the estimation of ASIR, stratified by gender, from 2021 through to the year 2036.

In all analytical processes, we used joinpoint (5.0.2) and R software (4.4.1) along with their respective packages.

## Results

### Descriptive analysis

#### Analysis of overall changes

Figuress 1,2 and Table 1 show the prevalence, incidence, deaths, DALYs number, and age-standardized rates of AF/AFL for the different age groups in 2021 for the global context and China. Globally, the prevalence of AF/AFL among all people increased by 137% between 1990 and 2021, from 22.2 million to 52.6 million. And it has decreased compared to 2019, which affected 60.0 million people worldwide. However, AF/AFL in 2021 affected 10.8 million people in China, an increase of 237% from 3.2 million. The age-standardized prevalence rate (ASPR) of AF/AFL increased by 14%, from 457 per 100,000 population in 1990 to 524 per 100,000 population in 2021 in China. Surprisingly, the aged-standardized rate of China's AF/AFL—related deaths actually decreased from 4.9 per 100,000 population in 1990 to 4.3 per 100,000 population in2021, while the global AF/AFL aged-standardized mortality rate (ASMR) was still slightly increasing from 4.2 per 100,000 population in 1990 to 4.3 per 100,000 population. It was mainly due to the significant reduction in female ASMR from 5.5 per 100,000 population in 1990 to 4.6 per 100,000 population, as male ASMR was still slightly increasing from 3.6 per 100,000 population in 1990 to 3.8 per 100,000 population in 2021. Despite the decline in female ASMR, the reduction trend in DALYs was not as pronounced as in males. The age-standardized DALYs in China decreased from 98 per 100,000 population in 1990 to 88 per 100,000 population in 2021 for females, while the males increased from 82 per 100,000 population to 90 per 100,000 population. Furthermore, it is noteworthy that it also demonstrated that the changes in ASIR, ASPR, ASMR, and age-standardized DALYs of AF/AFL globally over the past 30-plus years have not been significant and tend to stabilize, with 95% UI all including 0, although the all all-age prevalence, incidence, deaths and DALYs increased. During this period, China's ASMR and age-standardized DALYs showed similar patterns of change as well. Table 1 presents a detailed analysis of the all-age population counts and age-standardized rates, segregated by gender, for both the global context and China specifically. It is evident that, both within the confines of China and from a worldwide viewpoint, the burden of disease among men is higher than that observed in women.

**Figure 1:**
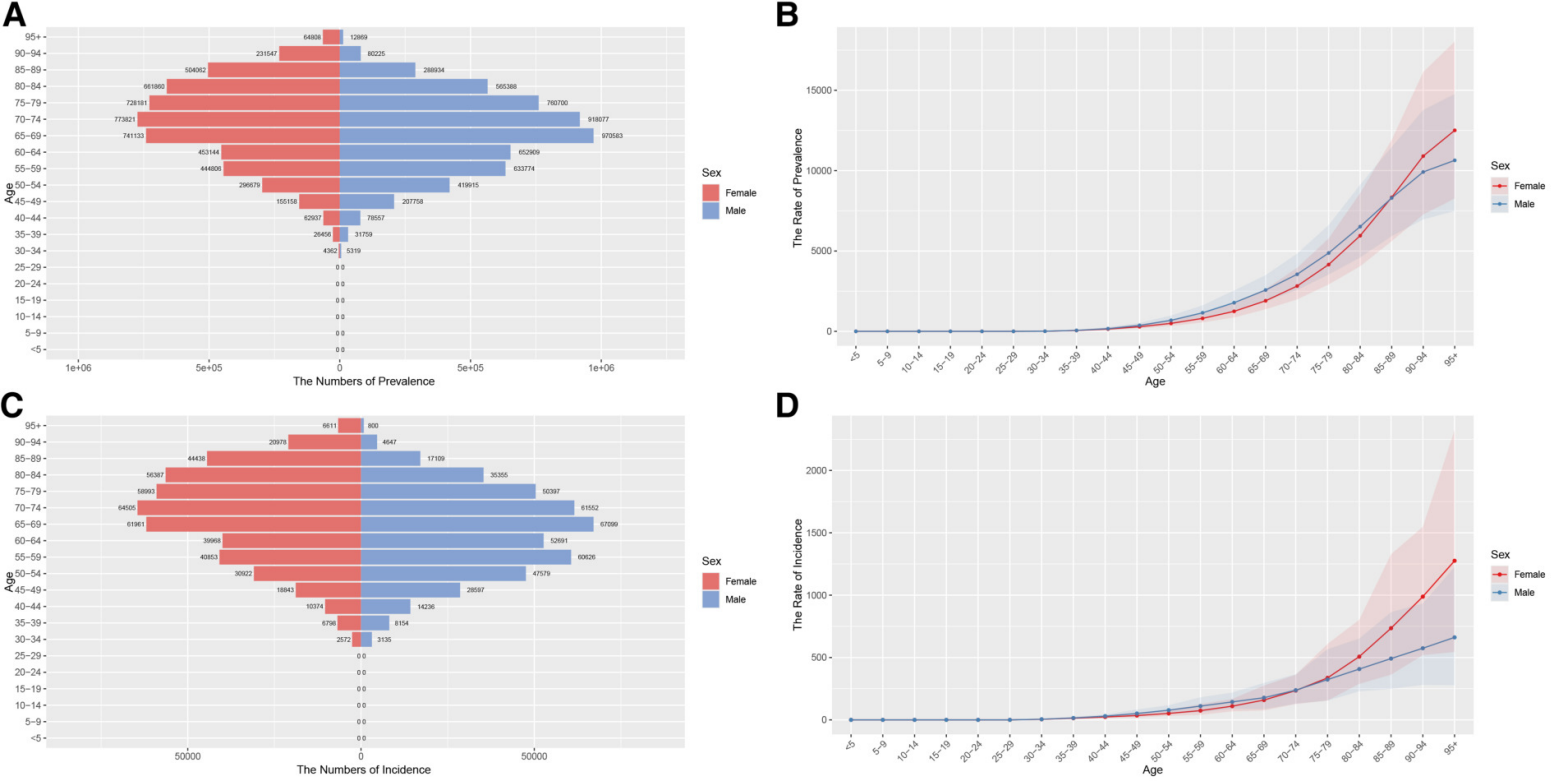
Aged-specific number and age-standardized prevalence and incidence rates of AF/AFL in China. **(A)** Aged-specific prevalence number. **(B)** Age-standardized prevalence rate. **(C)** Aged-specific incidence number. **(D)** Age-standardized incidence rate.

**Figure 2:**
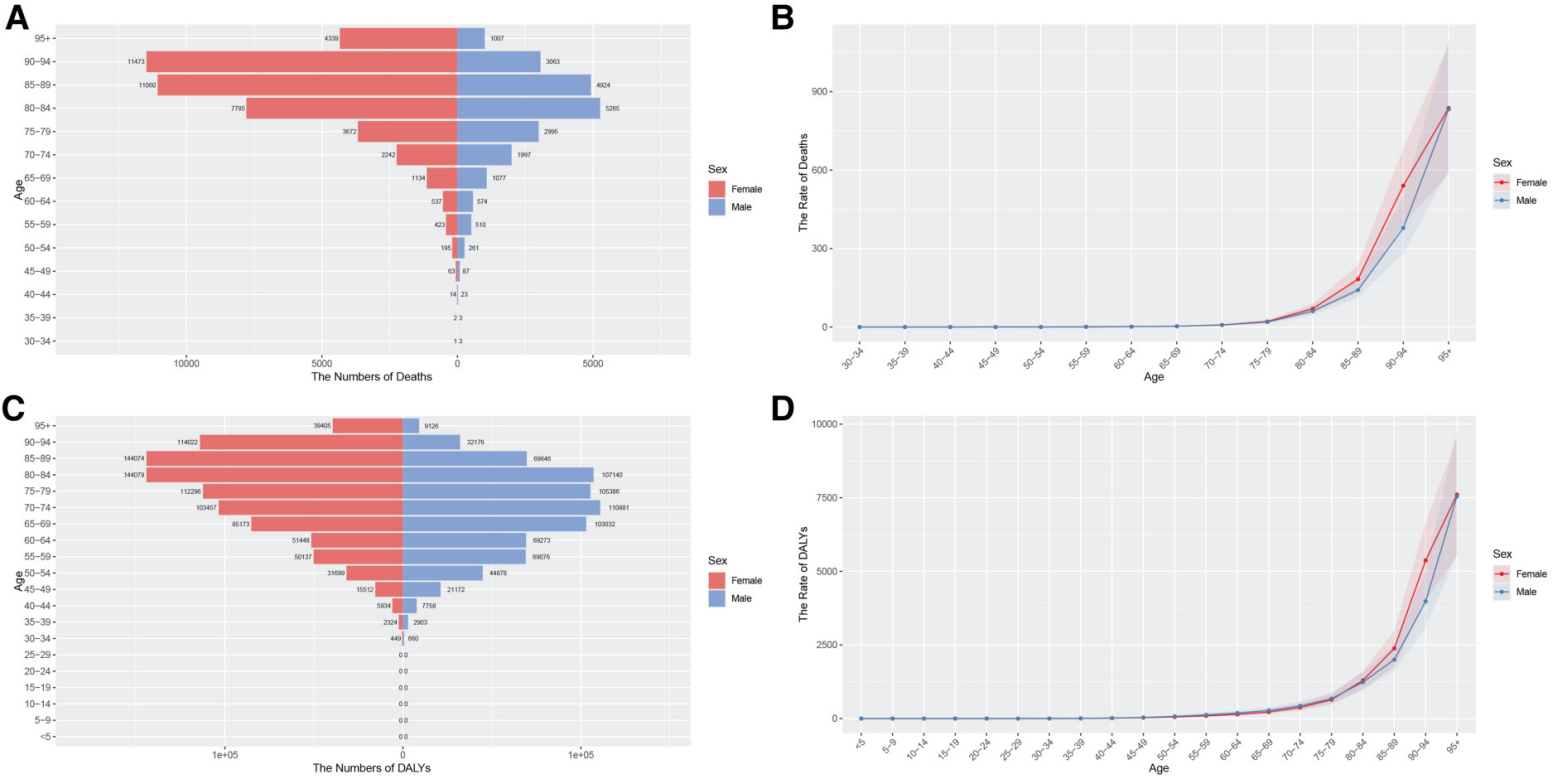
Aged-specific number and age-standardized mortality and DALYs rates of AF/AFL in China. **(A)** Aged-specific mortality number. **(B)** Age-standardized mortality rate. **(C)** Aged-specific DALYs number. **(D)** Age-standardized DALYs rate.

**Table 1:** All-age cases and age-standardized prevalence, incidence, deaths, and DALYs rates in 2021 for AF/AFL in China and worldwide.

	Prevalence (95% UI)			Incidence (95% UI)		
	2021 counts	2021 ASR per 100,000 people	Percentage change in ASR, 1990–2021	2021 counts	2021 ASR per 100,000 people	Percentage change in ASR, 1990–2021
Global						
Sex						
Male	27,899,046 (23026328,34646024)	728.88 (601.91,895.81)	0 (−0.04,0.06)	2,295,811 (1853679,2899436)	57.11 (46.19,72.14)	−0.02 (−0.05,0.04)
Female	24,65,2999 (20031485,30931399)	529.12 (430.79,663.14)	0 (−0.04,0.05)	21,89,116 (1723110,2821648)	47.26 (37.38,60.87)	0 (−0.04,0.05)
Total	52,55,2045 (43137876,64963854)	620.51 (511.36,768.88)	0.01 (−0.03,0.06)	44,84,926 (3610620,5706019)	52.12 (41.85,66.23)	−0.01 (−0.04,0.04)
China						
Sex						
Male	56,26,767 (4446323,7289510)	574.5 (456.9,745.56)	0.18 (0.13,0.24)	451,977 (348036,591930)	45.23 (35.38,59.31)	0.08 (0.03,0.13)
Female	51,48,954 (4069341,6741869)	473.4 (373.44,613.68)	0.10 (0.07,0.14)	464,203 (351459,620287)	43.28 (32.9,57.37)	0.04 (0.01,0.07)
Total	10,77,5721 (8531627,14014036)	524 (418.15,681.23)	0.14 (0.11,0.19)	916,180 (707384,1201381)	44.92 (34.96,59.42)	0.05 (0.01,0.09)
	Deaths (95% UI)			DALYs (95%)		
	2021 counts	2021 ASR per 100,000 people	Percentage change in ASR, 1990–2021	2021 counts	2021 ASR per 100,000 people	Percentage change in ASR, 1990–2021
Global						
Sex						
Male	134,700 (120296,145862)	4.44 (3.94,4.81)	0.06 (−0.04,0.20)	40,32,121 (3310673,4901830)	112.05 (93.3,135.28)	0.02(−0.04,0.10)
Female	204,247 (167703,228405)	4.29 (3.53,4.8)	0.01 (−0.09,0.12)	4,326,773 (3603522,5211317)	92.24 (76.84,111.24)	−0.01(−0.08,0.06)
Total	338,947 (288954,368613)	4.36 (3.69,4.75)	0.03 (−0.06,0.12)	83,58,894 (6970688,10133489)	101.4 (84.89,122.41)	0.01 (−0.05,0.07)
China						
Sex						
Male	21,789 (16730,28054)	3.81 (2.99,4.8)	0.07 (−0.21–0.69)	753,106 (569017,959037)	89.62 (70.01,111.29)	0.09 (−0.09,0.40)
Female	42,939 (32263,54478)	4.58 (3.45,5.83)	−0.17 (−0.41–0.14)	900,010 (695765,1116492)	87.68 (67.72,108.49)	−0.10 (−0.29,0.10)
Total	64,728 (51765,77729)	4.33 (3.43,5.23)	−0.12 (−0.35,0.16)	16,53,117 (1303681,2056459)	89.76 (72.13,109.67)	−0.04 (−0.19,0.14)

#### Analysis of age group in China

Figures 1, 2 illustrate the prevalence, incidence, mortality, and DALYs associated with AF/AFL across different age groups for both males and females in China for the years 1990 and 2021. Conspicuously, the age group of 64–74 years has the highest proportion of both incidence and prevalence cases (Figures 1A,C). After standardization for age, the ASPR and ASIR of AF/AFL in females aged 40–74 are notably lower than those among males. However, after around 89 years of age, the ASPR in females begins to exceed that of men. Additionally, after the age of 74, the ASIR in females also starts to surpass that of men. Furthermore, both the ASPR and ASIR of AF/AFL exhibit an upward trend with advancing age (Figures 1B,D). It is apparent that beginning at the age of 70, a substantial difference in ASMR becomes evident between the sexes, with women exhibiting superior performance compared to men. However, as age advances, this gap is continuously narrowing (Figures 2A,B). There aren't pronounced differences in age-standardized DALYs between males and females from 30 to 84 years of age. However, beyond this age range, the age-standardized DALYs in females commence to surpass males and this gap also narrows with the increase in age (Figures 2C,D).

**Figure 3:**
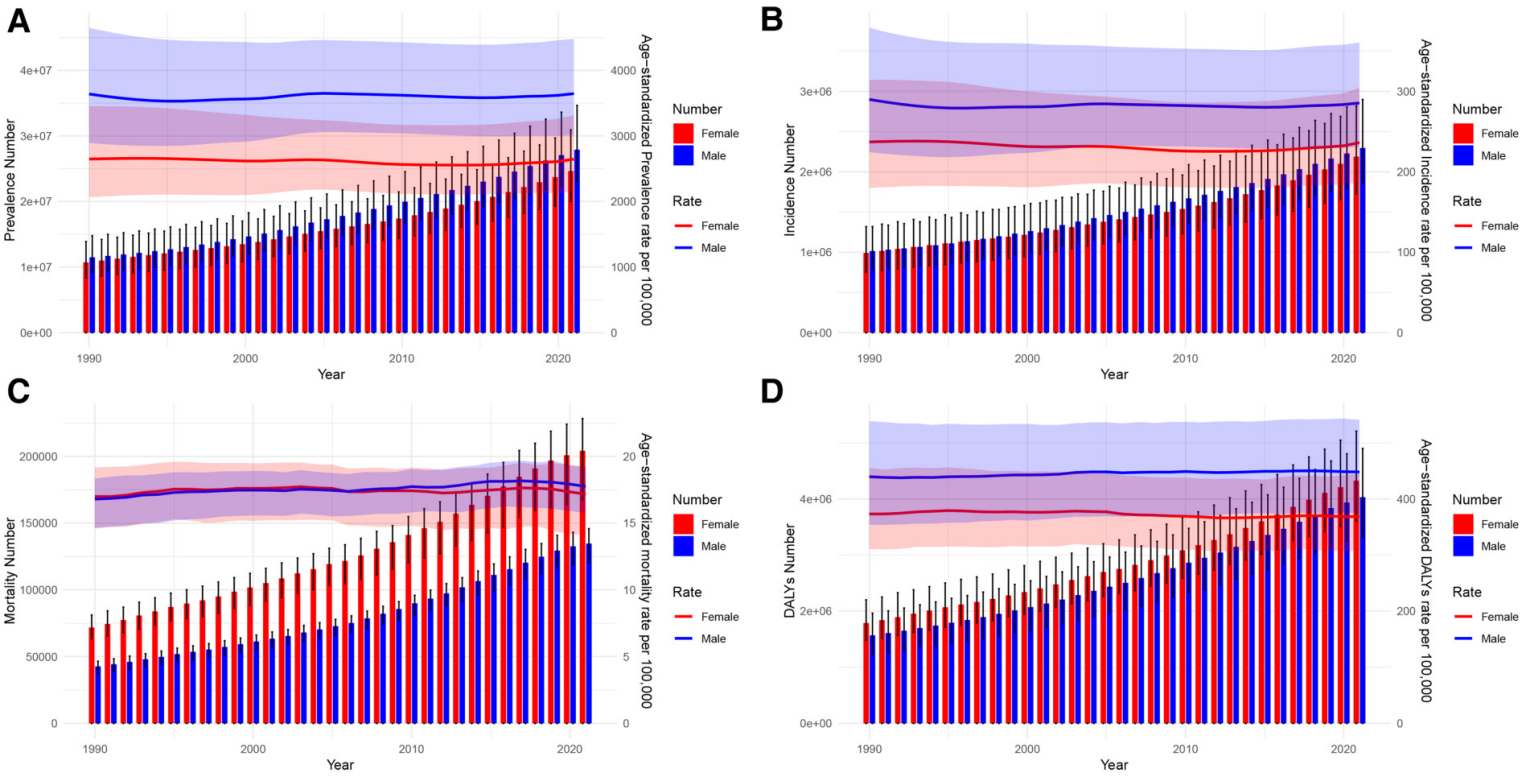
Global trends in the all-age cases and age-standardized prevalence and incidence rates of AF/AFL by sex from 1990 to 2021. **(A)** Prevalence number and rate. **(B)** Incidence number and rate. **(C)** Mortality number and rate. **(D)** DALYs number and rate.

**Figure 4:**
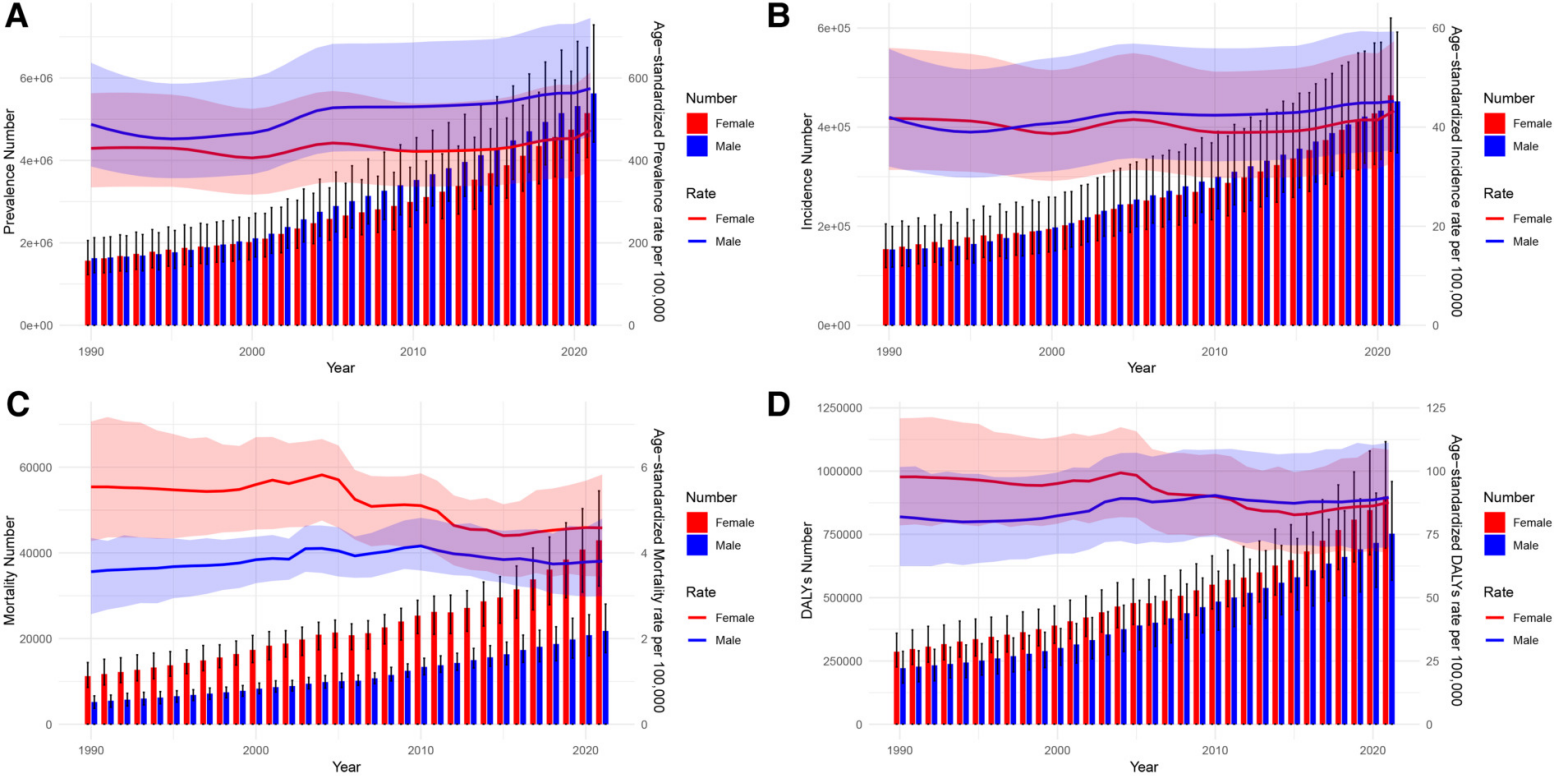
Trends of China in the all-age cases and age-standardized prevalence and incidence rates of AF/AFL by sex from 1990 to 2021. **(A)** Prevalence number and rate. **(B)** Incidence number and rate. **(C)** Mortality number and rate. **(D)** DALYs number and rate.

From 1990 to 2021, Figures 3, 4 illustrate the evolving trends in the sex-specific, all-age number, and age-standardized rates of AF/AFL prevalence, incidence, mortality, and disability-adjusted life years (DALYs), both globally and specifically within the context of China. It can be seen that globally, the sex-specific ASIR, ASPR, ASMR, and age-standardized DALYs have not shown significant increases or decreases, maintaining a stable trend (Figure 3). In recent years, the ASIR and ASPR of females in China have been higher than males, just like globally, and the trend has not been significantly changing (Figures 4A,B). Surprisingly, in China, the female ASMR is always higher than males from 1990 to 2021, but there is no such trend globally (Figures 3C, 4C). In recent years, the ASMR among males has been slightly higher than that of females, with little difference globally (Figure 3C). Furthermore, it is noteworthy that while females in China exhibit a higher ASMR compared to males, their age-standardized DALYs have consistently decreased below those of males since 2010, marking a difference from the global trend. From a global perspective, the age-standardized DALYs for females are still higher than those for males (Figures 3D, 4D).

### Joinpoint regression analysis

**Figure 5:**
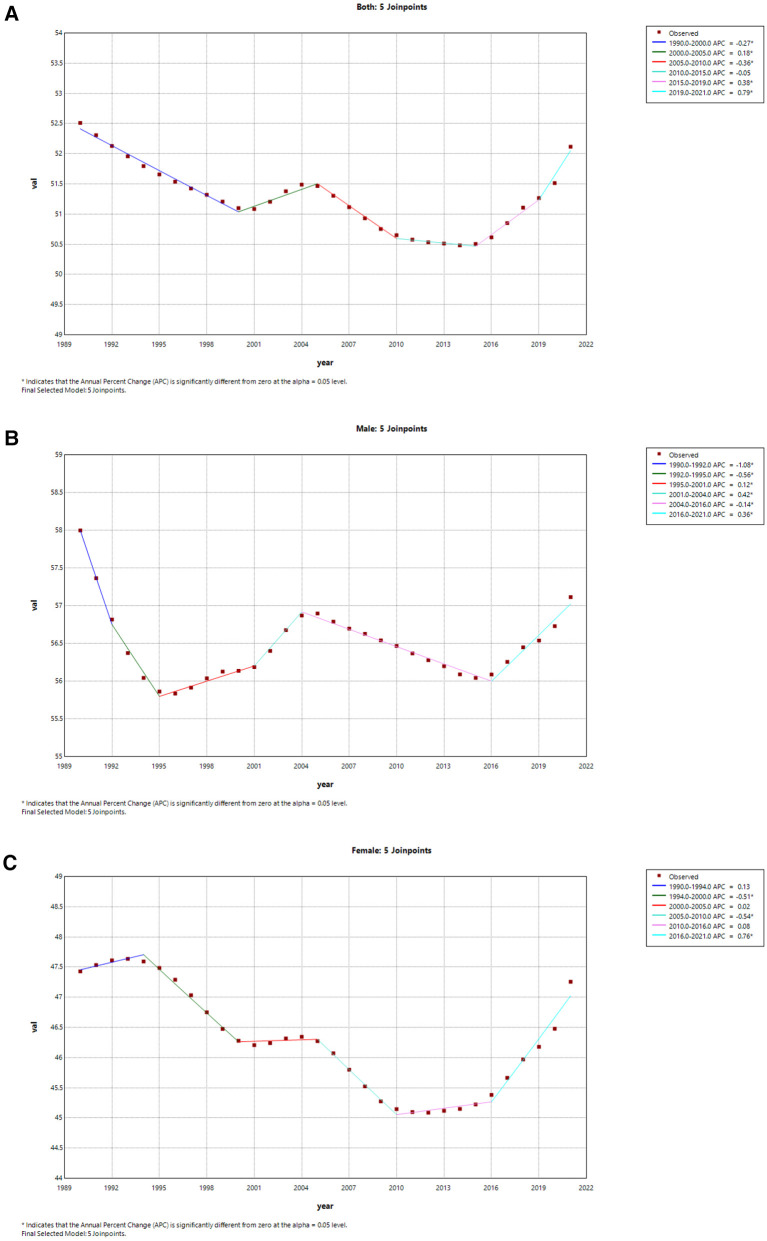
Joinpoint regression analysis of the sex-specific age-standardized incidence rate for AF/AFL worldwide from 1990 to 2021. **(A)** Age-standardized incidence rate for both. **(B)** Age-standardized incidence rate for males. **(C)** Age-standardized incidence rate for females.

**Figure 6:**
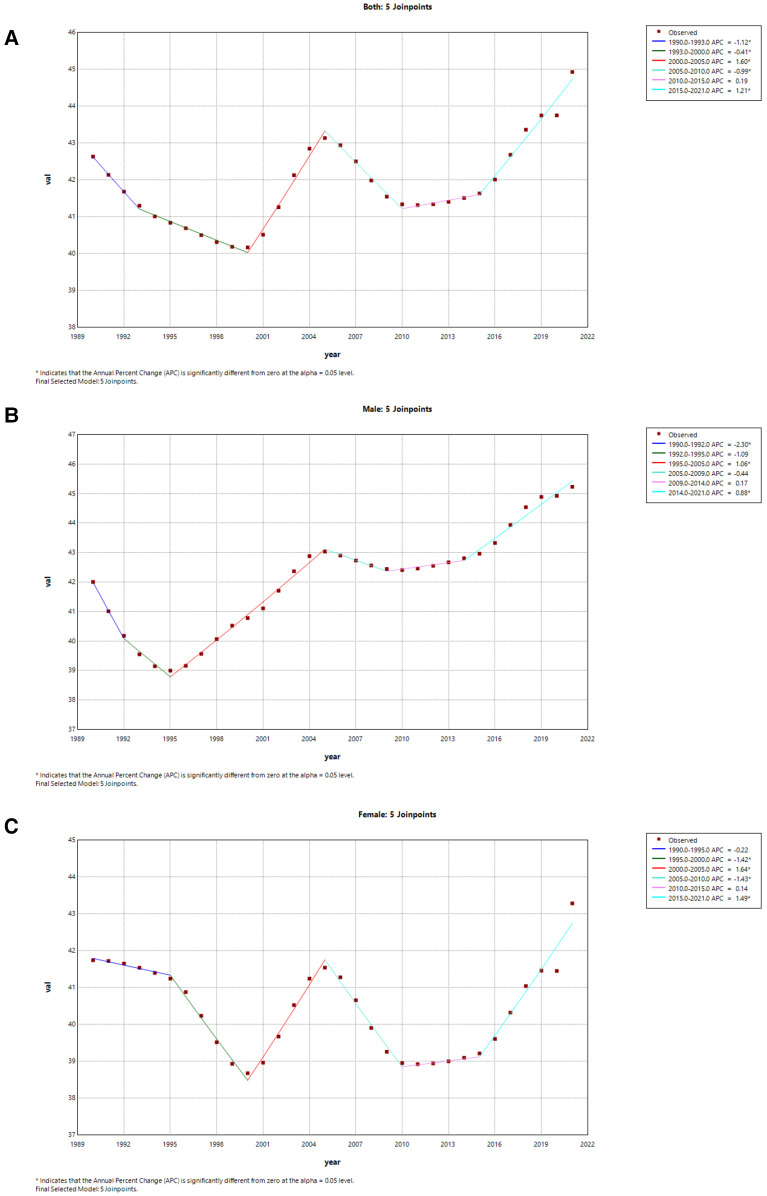
Joinpoint regression analysis of the sex-specific age-standardized incidence rate for AF/AFL in China from 1990 to 2021. **(A)** Age-standardized incidence rate for both. **(B)** Age-standardized incidence rate for males. **(C)** Age-standardized incidence rate for females.

Tables 2, 3 present the AAPCs and APCs analyses for AF/AFL incidence and mortality rates in China over the past 30-plus years, as well as in a global context, respectively. Globally, we observed a decrease in ASIR of AF/AFL in both sexes from 1990 to 2015, despite a slight increase between 2000 and 2005 (APC = 0.18), and a significant decline occurred between 2005 and 2010 (APC = −0.36) (Figure 5A). There was an increase in ASIR from 2015 to 2021 in both males and females (2015–2019 APC = 0.38, 2019–2021 APC = 0.79) (Figure 5A). However, we found a slight increase in ASIR from 1990 to 2021 in both sexes in China (AAPC = 0.16 95% CI: 0.05,0.26), while a pronounced increase appeared from 2000 to 2005 (APC = 1.60), and a marked decrease occurred from 1990 to 1993 (APC = −1.12) (Figure 6A). The male ASIR has a notable increase (AAPC = 0.25 95% CI: 0.10, 0.40) compared to the female, and the fluctuation of female ASIR is very large, with no significant trend of change (AAPC = 0.07 95% CI: −0.01, 0.24; *p* > 0.05) (Figures 6B,C).

**Table 2:** Joinpoint regression analysis: trends in age-standardized incidence, mortality rates (per 100,000 persons) among both sexes, males, and females in worldwide, 1990–2021.

Gender	ASIR			ASMR		
	Period	APC (95% CI)	AAPC (95% CI)	Period	APC (95% CI)	AAPC (95% CI)
Both	1990–2000	−0.26 (−0.28—−0.25)*	−0.02 (−0.05–0.00)	1990–1995	0.66 (0.52–0.81)*	0.11 (0.03–0.18)*
	2000–2005	0.18 (0.12–0.25)*		1995–2003	0.16 (0.08–0.24)*	
	2005–2010	−0.35 (−0.41—−0.29)*		2003–2006	−0.40 (−0.98–0.18)	
	2010–2015	−0.05 (−0.11–0.01)		2006–2012	0.06 (−0.07–0.19)	
	2015–2019	0.38 (0.28–0.48)*		2012–2018	0.36 (0.22–0.51)*	
	2019–2021	0.79 (0.59–0.99)*		2018–2021	−0.88 (−1.22—−0.54)*	
Male	1990–1994	0.13 (−0.07–0.33)	−0.05 (−0.09—−0.01)*	1990–1997	0.55 (0.45–0.65)*	0.18 (0.09–0.27)*
	1994–2000	−0.51 (−0.65—−0.37)*		1997–2006	0.02 (−0.05–0.09)	
	2000–2005	0.02 (−0.16–0.19)		2006–2012	0.25 (0.11–0.39)*	
	2005–2010	−0.54 (−0.71—−0.38)*		2012–2015	0.77 (0.13–1.42)*	
	2010–2016	0.08 (−0.04–0.20)		2015–2019	−0.09 (−0.40–0.23)	
	2016–2021	0.76 (0.64–0.89)*		2019–2021	−0.92 (−1.57—−0.27)*	
Female	1990–1992	−1.08 (−1.35—−0.80)*	−0.03 (−0.09–0.03)	1990–1995	0.64 (0.46–0.83)*	0.05 (−0.05–0.15)
	1992–1995	−0.56 (−0.83—−0.29)*		1995–2003	0.16 (0.05–0.26)*	
	1995–2001	0.12 (0.06–0.18)*		2003–2006	−0.53 (−1.28–0.23)	
	2001–2004	0.42 (0.18–0.66)*		2006–2013	−0.08 (−0.22–0.05)	
	2004–2016	−0.14 (−0.15—−0.12)*		2013–2018	0.40 (0.13–0.67)*	
	2016–2021	0.36 (0.31–0.42)*		2018–2021	−0.93 (−1.40—−0.47)*	

*Indicates *p* value < 0.05.

**Table 3:** Joinpoint regression analysis: trends in age-standardized incidence, mortality rates (per 100,000 persons) among both sexes, males, and females in China, 1990–2021.

Gender	ASIR			ASMR		
	Period	APC (95% CI)	AAPC (95% CI)	Period	APC (95% CI)	AAPC (95% CI)
Both	1990–1993	−1.12 (−1.62—−0.61)*	0.16 (0.05–0.26)*	1990–1998	−0.11 (−0.42–0.21)	−0.45 (−0.78—−0.12)*
	1993–2000	−0.41 (−0.59—−0.24)*		1998–2004	1.41 (0.91–1.92)*	
	2000–2005	1.60 (1.27–1.92)*		2004–2007	−3.78 (−5.61—−1.90)*	
	2005–2010	−0.99 (−1.30—−0.68)*		2007–2010	0.67 (−1.21–2.57)	
	2010–2015	0.19 (−0.13–0.50)		2010–2013	−4.12 (−6.10—−2.11)*	
	2015–2021	1.21 (1.04–1.38)*		2013–2021	0.06 (−0.23–0.35)	
Male	1990–1992	−2.30 (−3.39—−1.20)	0.25 (0.10–0.40)*	1990–2001	0.70 (0.54–0.86)*	0.18 (−0.11–0.47)
	1992–1995	−1.09 (−2.19–0.02)*		2001–2004	2.29 (0.68–3.92)*	
	1995–2005	1.06 (0.96–1.16)*		2004–2007	−1.26 (−2.86–0.38)	
	2005–2009	−0.44 (−0.97–0.10)*		2007–2010	1.74 (0.04–3.46)*	
	2009–2014	0.17 (−0.17–0.51)		2010–2014	−1.83 (−2.76—−0.90)*	
	2014–2021	0.88 (0.73–1.02)*		2014–2021	−0.41 (−0.72—−0.09)*	
Female	1990–1995	−0.22 (−0.59–0.16)*	0.07 (−0.10–0.24)	1990–1998	−0.27 (−0.63–0.10)	−0.64 (−1.04—−0.23)*
	1995–2000	−1.42 (−1.94—−0.90)		1998–2004	1.19 (0.60–1.78)*	
	2000–2005	1.64 (1.12–2.17)*		2004–2007	−4.49 (−6.71—−2.21)*	
	2005–2010	−1.43 (−1.93—−0.93)		2007–2010	0.37 (−1.92–2.71)	
	2010–2015	0.14 (−0.37–0.65)		2010–2013	−4.50 (−6.91—−2.04)*	
	2015–2021	1.49 (1.21–1.77)*		2013–2021	0.22 (−0.13–0.57)	

* Indicates *p* value < 0.05.

**Figure 7:**
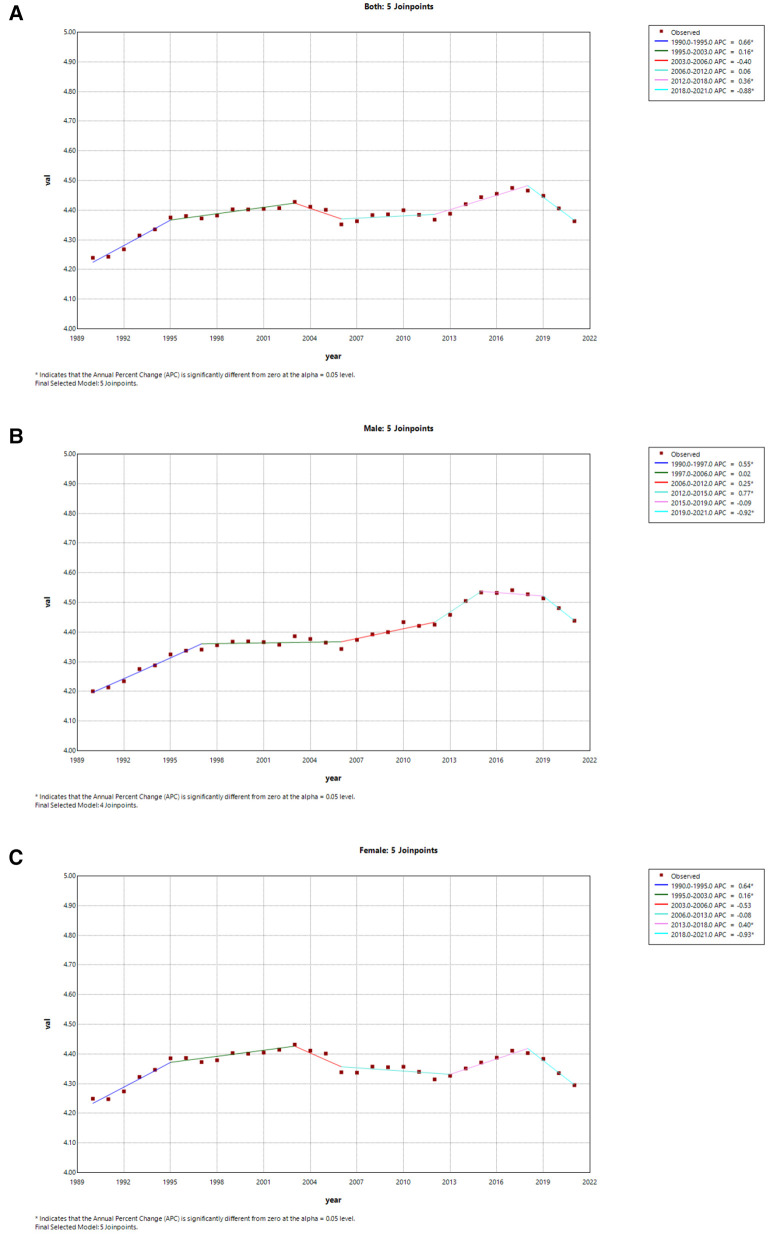
Joinpoint regression analysis of the sex-specific age-standardized mortality rate for AF/AFL worldwide from 1990 to 2021. **(A)** Age-standardized mortality rate for both. **(B)** Age-standardized mortality rate for males in worldwide. **(C)** Age-standardized mortality rate for females in worldwide.

**Figure 8:**
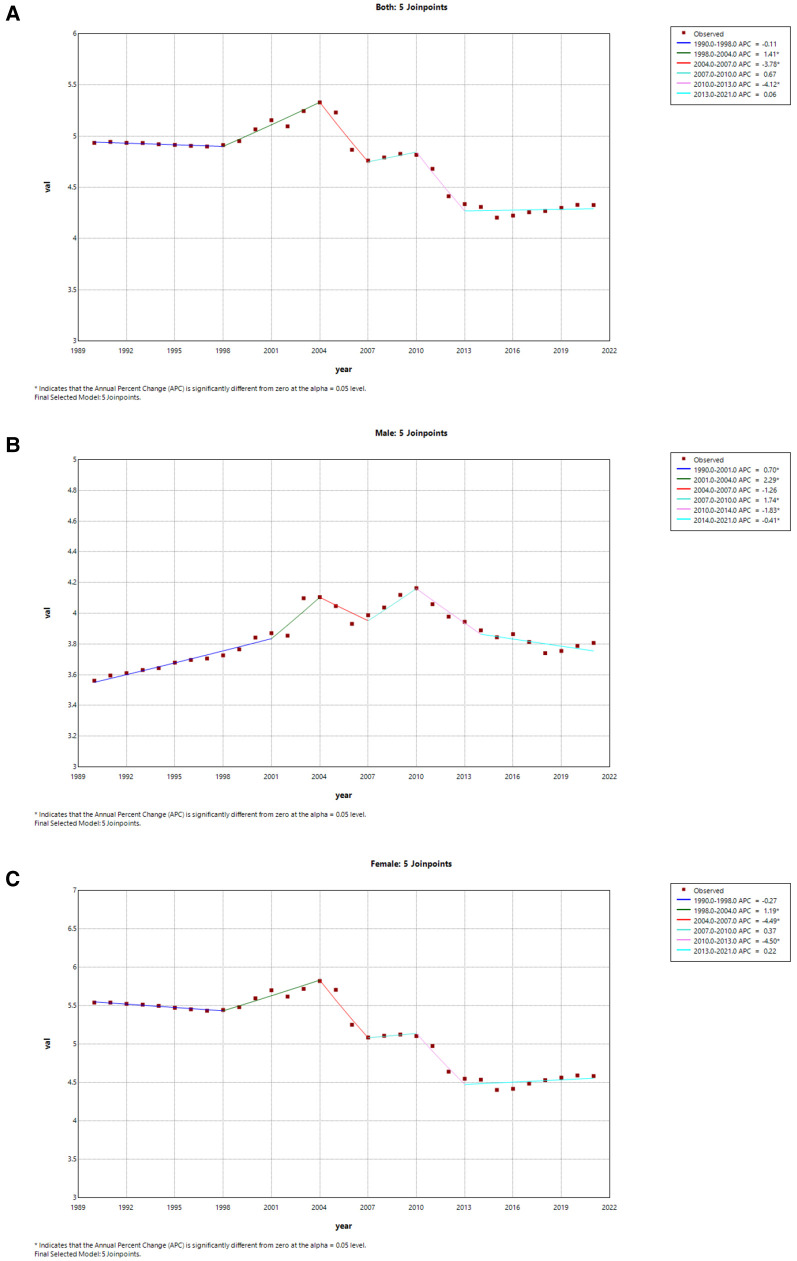
Joinpoint regression analysis of the sex-specific age-standardized mortality rate for AF/AFL in China 1990 to 2021. **(A)** Age-standardized mortality rate for both. **(B)** Age-standardized mortality rate for males. **(C)** Age-standardized mortality rate for females.

From a global perspective, only male ASMR in both sexes has shown a significant upward trend from 1990 to 2021 (AAPC = 0.19 95% CI: 0.09, 0.30) (Figure 7A). However, the ASMR in both sexes has a decrease from 1990 to 2021 in China (AAPC = −0.45 95% CI: −0.78, −0.12), and the marked decline was from 2010 to 2013 (APC = −4.12) (Figure 8A). Additionally, the significant decline of China in female ASMR occurred from 2010 to 2014 (APC = −1.83), while the most pronounced decrease in male ASMR appeared from 2010 to 2013 (APC = −4.50) (Figures 8B,C).

### The analysis of age, period, and cohort on ASIR and ASMR in China

**Figure 9:**
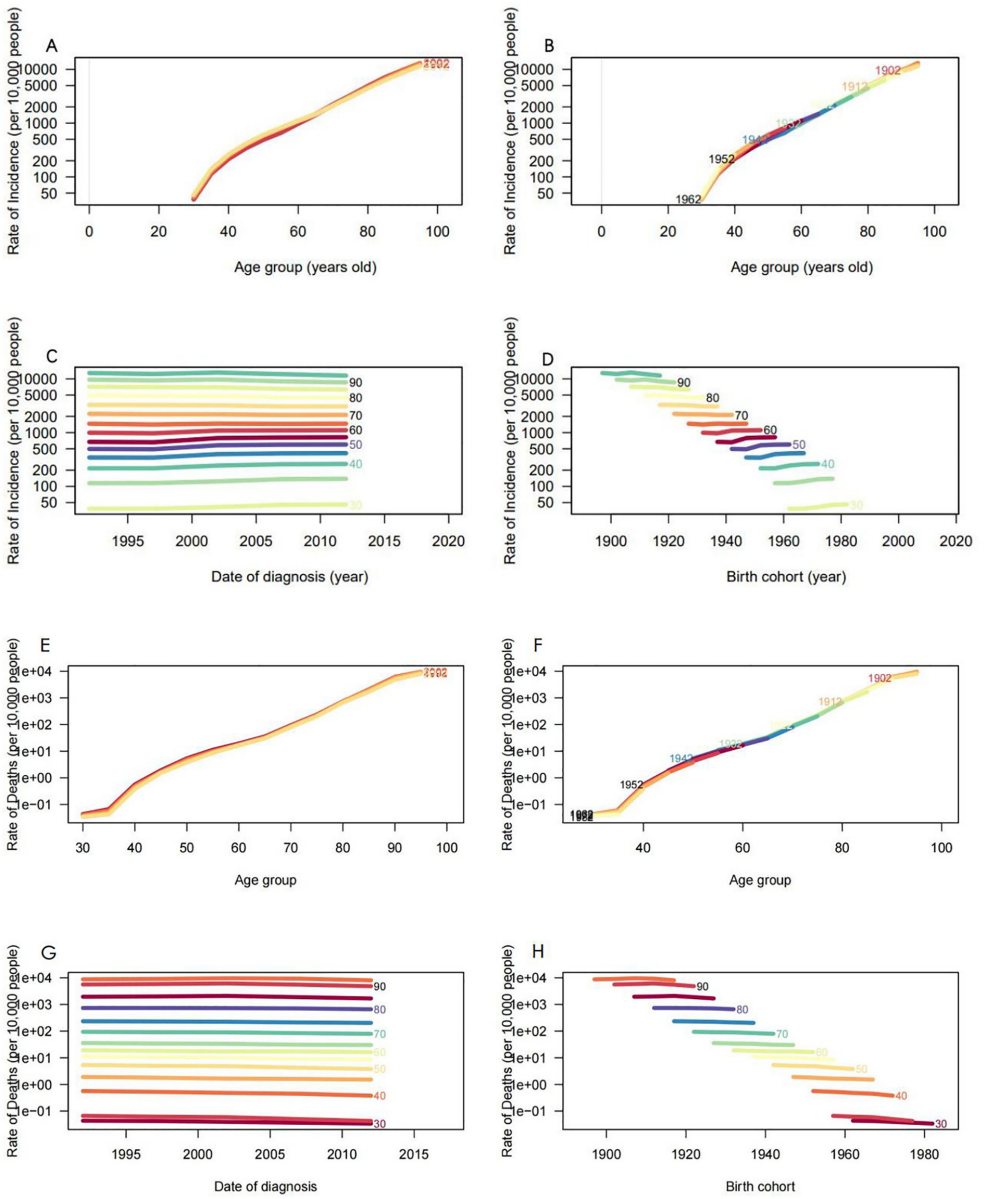
Incidence and mortality rates of AF/AFL in China. **(A)** The age-specific incidence rates of AF/AFL according to time periods; each line connects the age-specific mortality for a 5-year period. **(B)** The age-specific incidence rates of AF/AFL according to birth cohorts; each line connects the age-specific mortality for a 5-year cohort. **(C)** The period-specific incidence rates of AF/AFL according to age groups; each line connects the birth cohort-specific mortality for a 5-year age group. **(D)** The birth cohort-specific incidence rates of AF/AFL according to age groups; each line connects the birth cohort-specific mortality for a 5-year age group. **(E)** The age-specific mortality rates of AF/AFL according to time periods; each line connects the age-specific mortality for a 5-year period. **(F)** The age-specific mortality rates of AF/AFL according to birth cohorts; each line connects the age-specific mortality for a 5-year cohort. **(G)** The period-specific mortality rates of AF/AFL according to age groups; each line connects the birth cohort-specific mortality for a 5-year age group. **(H)** The birth cohort-specific mortality rates of AF/AFL according to age groups; each line connects the birth cohort-specific mortality for a 5-year age group.

Figures 9A,E depict the incidence and mortality trends of AF/AFL across different age brackets for the years 1992, 1996, 2000, 2004, 2008, 2012, and 2017. Significantly, both incidence and mortality rates escalate sharply with advancing age. Figures 9B,F clearly illustrate the cohort-specific patterns in the occurrence and fatality rates of AF/AFL, emphasizing the differences across various birth cohorts. Figures 9C,G present the long-term trends in AF/AFL incidence and mortality from 1990 to 2021, revealing a notable increase in incidence rates from the age of 30 to 60, followed by a decline post age 70. In contrast, mortality rates exhibit a subtle downward trend across all age groups. The incidence and mortality rates are higher among the elderly, while they are markedly lower in younger populations. Figures 9D,H clearly illustrate how the incidence and mortality rates have changed over time for different age groups, providing valuable insights into the variations across various birth cohorts. In younger ages, the incidence rate rises with each successive year of birth, whereas in older ages, it declines. The mortality rate, however, consistently displays a decreasing trend across all age groups with each subsequent year of birth. Ultimately, when considering the collective influence of age, period, and cohort, the comprehensive model emerges as the most effective in capturing the individual contributions of these factors.

### Decomposition analysis in China

**Figure 10:**
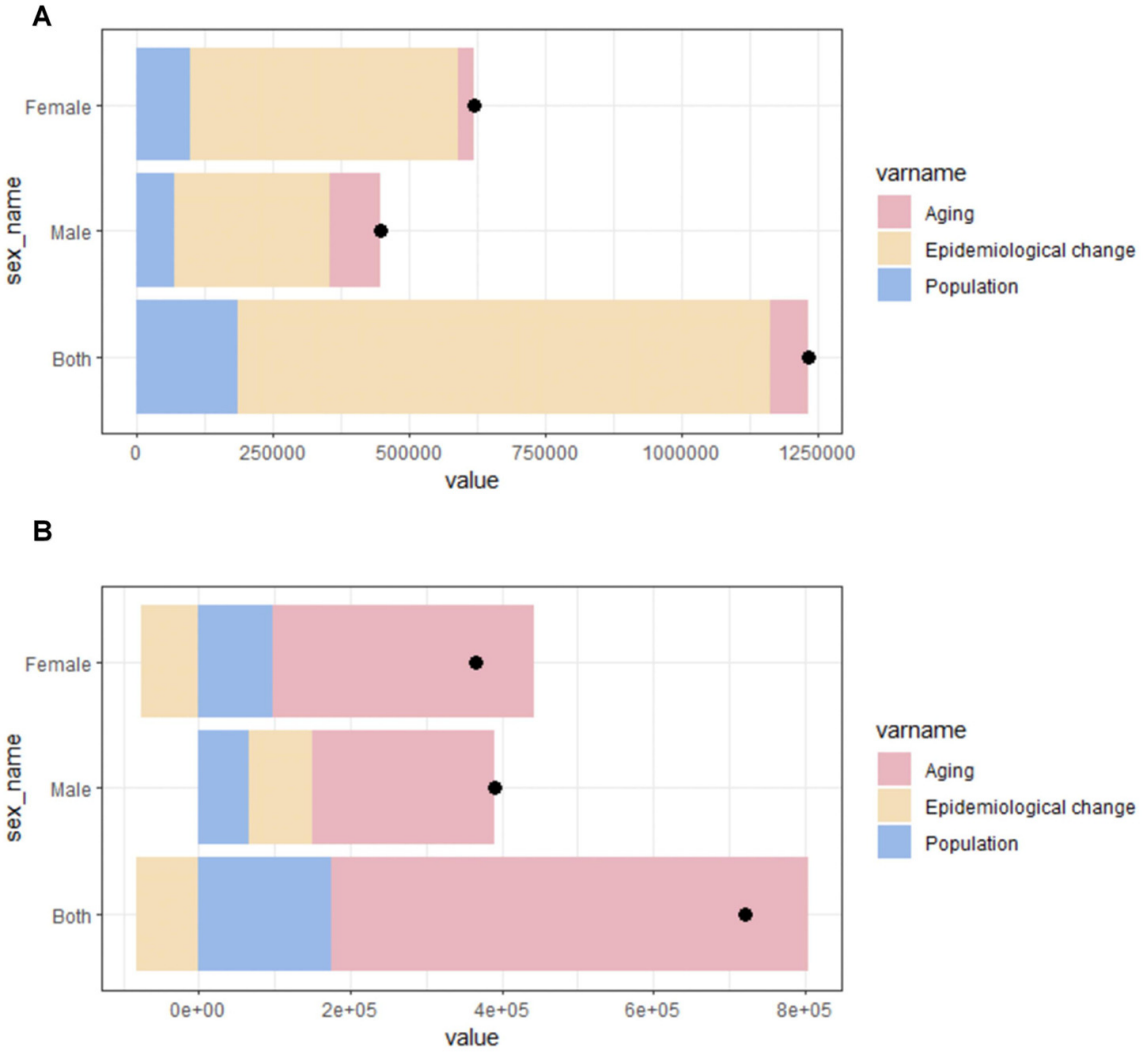
Changes in AF/AFL incidence **(A)** and mortality **(B)** according to population growth, aging, and epidemiological change from 1990 to 2021 at the China level and by gender. The black dot represents the overall value of change contributed by all 3 components.

To investigate the comprehensive impact of aging, population dynamics, and epidemiological changes on the epidemiology of AF/AFL, we performed a meticulous decomposition analysis of case counts, considering variables such as population, the aging, and epidemiological changes (Figure 10). It is observed that epidemiological change contributed 79.05% to the increased incidence of AF/AFL incidence between 1990 and 2021, the it is also the highest percentage among males and females respectively (Table 4). Although the proportion of aging in both sexes is not very large, the proportion of aging among males (20.59%) has significantly increased in comparison to females (4.59%). As for the mortality, aging growth accounted for 87.28% in both sex of the increase, which is also the pronounced upward trend. In the meantime, the contribution of aging to overall mortality is most significant in females (94.26%). The effect of epidemiological change on mortality growth was negative (−11.54%) in both sexes, and this effect was the most pronounced in males (−21.14%). Conversely, the effect of epidemiological change on mortality growth was positive in males (21.18%). The effects of demography and epidemiology on incidence and mortality differed across sex.

**Table 4:** Decomposition analysis: changes in incidence and deaths number according to population-level determinants and causes from 1990 to 2021.

Measure	Overll difference	Change due to Population-level determinants (% contribute to the total changes)		
		Aging	Population	Epidemiological change
Incidence				
Both	12,31,791	69958.282 (5.68%)	1,88,125.609 (15.27%)	9,73,707.282 (79.05%)
Male	4,47,880	92227.802 (20.59%)	72016.107 (16.08%)	2,83,635.692 (63.33%)
Female	6,19,610	30685.098 (4.95%)	99470.49 (16.05%)	4,89,454.694 (78.99%)
Deaths				
Both	7,22,216	630382.09 (87.28%)	175194.104 (24.26%)	−83360.39 (−11.54%)
Male	3,90,113	241290.484 (61.85%)	66194.78 (16.97%)	82627.754 (21.18%)
Female	3,64,999	344062.617 (94.26%)	98106.638 (26.88%)	−77170.238 (−21.14%)

### Cross-country inequality analysis

**Figure 11:**
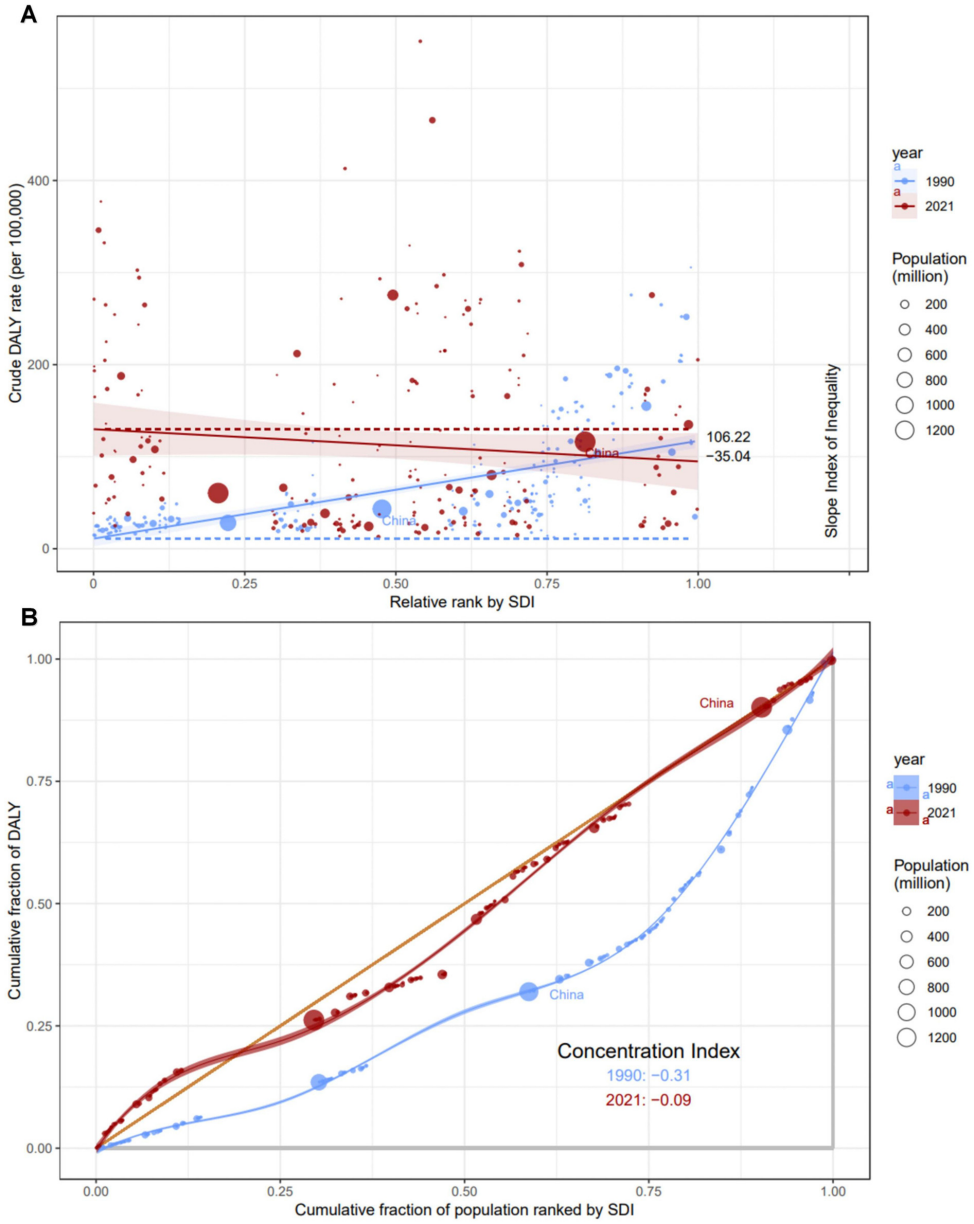
Health inequality regression curves **(A)** and concentration **(B)** for the DALYs of AF/AFL from 1990 to 2021 across the world.

Significant inequality in both overall and comparative SDI-related inequalities have been observed with respect to the burden of AF/AFL, accompanied by notable upwards in these indicators over time (Figure 11). Contrary to expectations, the prevalence of AF/AFL was higher in countries with higher SDI in 1990, yet by 2021, this trend reversed, with higher SDI countries exhibiting lower prevalence rates. The SII in 1990 stood at 106.22 per 100,000 population, signifying a marked excess prevalence in the most affluent nations compared to their least affluent counterparts. However, this disparity has significantly contracted, with the SII reaching −35.04 per 100,000 population by 2021, indicating a narrowing gap in disease prevalence between countries of varying SDI. This shift underscores the dynamic nature of global health disparities and the evolving impact of SDI factors on the distribution of non-communicable diseases like AF/AFL (Figure 11A). This indicates that there is a negative correlation between DALYs and SDI at present. This significant decline suggests that the inequality in age-standardized AF/AFL burden between low-income and high-income countries during this period has decreased. Meanwhile, the concentration index (CI) also showed an increase from 1990 (CI = −0.31) to 2021 (CI = −0.08) (Figure 11B). Although the inequality in AF/AFL burden between poor and rich countries within the region has decreased, inequality still persists. This indicates that, despite the narrowing of the wealth disparity in certain regions, the persistent global inequality within AF/AFL continues to pose a long-standing challenge.

### Predictive analysis

**Figure 12:**
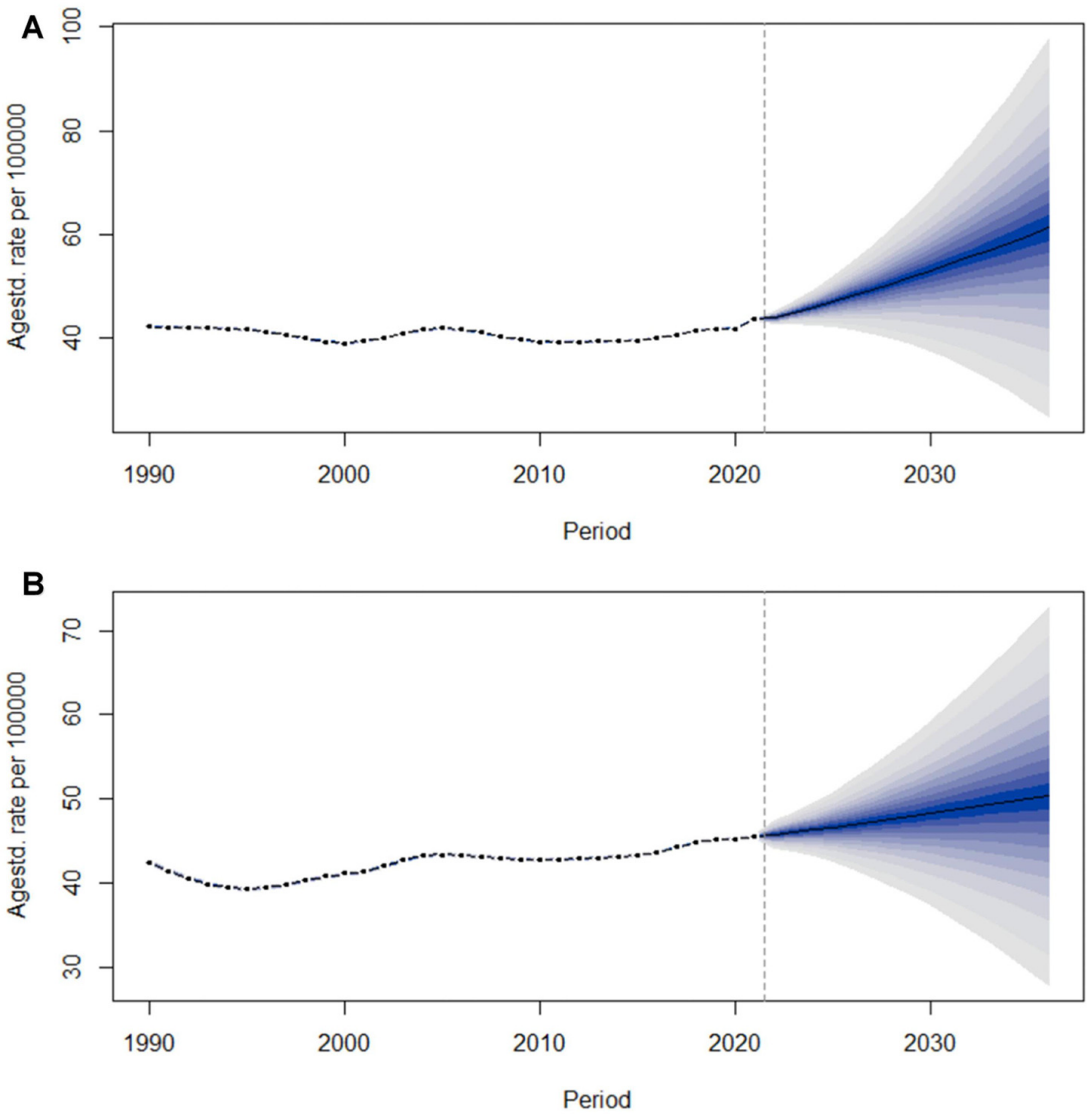
Trends of age-standardized incidence rate (ASIR) from 2021 to 2036 in females **(A)** and males **(B)** predicted by Bayesian age-period-cohort (BAPC) models.

To gain insights into the post-2021 trajectories of the Age-Standardized Incidence Rate (ASIR) for AF/AFL, we deployed Bayesian Age-Period-Cohort models to project the ASIR from 2021 to 2036, with data stratified by sex. As illustrated in Figure 10B, the ASIR for males is projected to escalate annually, rising from 45.5 per 100,000 in 2021 to 50.3 per 100,000 by 2036 (Figure 12B). Concurrently, females exhibit a similar upward trend, with the ASIR increasing from 43.7 per 100,000 in 2021 to a projected 61.2 per 100,000 by 2036 (Figure 12A). These projections indicate that the trends of ASIR in both males and females will experience an upward trajectory.

## Discussion

Over recent years, as management strategies for AF/AFL have been further refined, the global disease burden associated with this condition has seen a reduction. Pharmacological interventions for atrial fibrillation have focused on two primary objectives: restoring sinus rhythm through cardioversion and controlling the ventricular rate. These have emerged as the cornerstones of treatment and are pivotal for ensuring the long-term survival of individuals with atrial fibrillation. In 2021, the Asia Pacific Heart Rhythm Society (APHRS) updated the management pathway for atrial fibrillation, summarized as the “ABC comprehensive management pathway”: A stands for stroke prevention through anticoagulation therapy; B stands for patient-centered, symptom-directed heart rate or rhythm control; C stands for cardiovascular risk and comorbidity management as well as lifestyle changes (39). When atrial fibrillation persists for more than 48 h, anticoagulation therapy is usually required before conversion to sinus rhythm to reduce the risk of stroke. Additionally, Each patient with atrial fibrillation has heterogeneous stroke risk, therefore, physicians need to assess the patient's stroke risk based on the CHA_2_DS_2_-VASc score. When the score for males is ≥2 or for females is ≥3, it is recommended to take oral anticoagulants (40). As medical diagnostic and therapeutic technologies continue to evolve, the diagnostic accuracy for AF/AFL has seen a significant enhancement (39). The new ESC guidelines indicate that an electrocardiogram (ECG) is required to establish a diagnosis of AF. The assessment must encompass a 12-lead ECG recording or a single-lead ECG tracing that lasts over 30 s, showcasing non-identifiable repetitive P waves and irregular RR intervals, provided atrioventricular conduction is not compromised (41). An AI-driven targeted screening method, which utilizes existing clinical data, enhances the detection rate of atrial fibrillation and has the potential to improve the efficacy of atrial fibrillation screening (42). With the increasing attention to atrial fibrillation and the continuous update of technologies for detecting atrial fibrillation, the ASPR for AF may therefore increase. The research investigates the trends in the burden of AF/AFL in China over the last 30-plus decades. Based on our present understanding, this study stands as the initial comprehensive examination of the epidemiological patterns associated with AF/AFL within China and across the international community, utilizing a unique combination of joinpoint analysis and the apc modeling framework. Based on GBD 2019, Dong et al. (5) published a worldwide study on AF/AFL, and Cheng et al. (13) updated it with GBD 2021. Over the last three decades, AF/AFL has emerged as a severe and potentially fatal condition, with its incidence rising considerably (3). The prompt treatment of this condition is a matter of significant importance. Within the scope of our research, we have delineated the most extensive and up-to-date assessment of the global impact of AF/AFL, while also underscoring the projected trajectories of AF/AFL occurrence in the forthcoming years. Over the past three decades, the economic landscape and social environment have undergone significant transformations (15). Meanwhile, the observed increase in the global prevalence of AF/AFL, with a 137% rise from 1990 to 2021, underscores the growing importance of these cardiovascular conditions in public health.

The apparently declining ASMR in China is indicative of positive outcomes, which points towards the management and treatment of AF/AFL are being improved. Nevertheless, this fact of it is not globally followed the health situation. This reveals the disparity of health resources allocation and subsequently, the quality of healthcare services across different regions. The noteworthy decrease in female ASMR in China, alongside the small rise in males, insinuates that particular measures pertaining to gender may be called into question for the realization of the disparities in the diseases. The more pronounced decline in ASMR among women may be attributed to the implementation of the CHA_2_DS_2_-VASc score in AF/AFL management (40). This scoring system has significantly enhanced the anticoagulation treatment rate in women by enabling more precise risk stratification. By systematically identifying high-risk patients, the CHA_2_DS_2_-VASc score has facilitated targeted therapeutic interventions, particularly in women who were previously under-treated. This improved risk assessment and subsequent management have likely contributed to the observed reduction in ASMR among female AF/AFL patients, highlighting the importance of evidence-based risk stratification tools in optimizing clinical outcomes. The Results of age-period-cohort (apc) model showed the interaction of these factors in epidemiology. In recent years, catheter ablation for AF/AFL has become increasingly mature. Atrioventricular nodal ablation provides ventricular rate control effectively. However, the ablation developed a dependency on pacing (40). A recent study has shown that both cryoballoon pulmonary vein isolation (CB PVI) and pulsed field ablation (PFA) are potent and highly efficient techniques (43). Moreover, when comparing PFA with CB PVI, it exhibits similar procedural efficacy but is linked to shorter procedure times and no incidence of phrenic nerve palsies. Importantly, the 12-month clinical success rates are positive and do not differ significantly between the two groups (3). With the continuous development of ablation technology in controlling heart rate, more and more people can benefit, and it has reduced the mortality to some extent and the risk of atrial fibrillation recurrence and decrease cardiovascular hospitalizations significantly.

Incidence increases with age is a typical finding, but then there is a decline phenomenon after 70 years which is rather different. It is hard to say whether that results from the cumulative influence of risk factors over time or the natural history of the disease. The period of time, age, and birth cohort aspects on incidence and mortality rates of males and females have not clear further signals that gender-specific strategies related to research and intervention.

Decomposition analysis in China illustrates that the epidemiological trend is the core factor in the surge of AF/AFL, which means that preventable risk factors remain the main stack. For the last few decades, the hasty urbanization in China has spurred the people's sedentary lifestyle and a lack of exercise, which could possibly lead to the occurrence of AF/AFL. Likewise, nutrition has become transformed, as it could have resulted in the rise of an unhealthy diet that is rich in fatty, salty, and sugary foods. These changes might all work together to contribute to the growing threats of AF/AFL by affecting the speed of cell division.Moreover, within clinical settings, physicians ought to select therapeutic regimens and monitoring metrics that align with the gender-specific attributes of their patients. This approach aims to enhance both the efficacy of treatments and the overall quality of life for those under care.

The disproportion in the scope of disease burden in the world shows a disturbing trend where there is an increase in the disparities in the pain of AF/AFL. Blushing out the differences between high and low SDI countries in relation to prevalence is a good step, but the fact that inequality is still a problem indicates that more equitable access to healthcare resources is necessary so that all divisions of the population reap the benefits of these advancements in AF/AFL management. Over the last thirty years, China as a developing country has made substantial progress. The wave of AF/AFL disease burdens in China presents an unusual scenario within the global context, potentially signaling a shift in the country's SDI and healthcare strategies. Drawing insights from the experiences of developed countries could aid in further refining the distribution of medical resources and bolstering disease prevention and control initiatives, thereby alleviating the burden of AF/AFL diseases.

Predictive analysis indicates that the age-standardized incidence rates (ASIR) for both men and women in China will show an upward trend from 2021 to 2036, and women are anticipated to experience a greater burden of AF/AFL compared to men in the future. Public health departments should strengthen prevention publicity and intervention measures for AF/AFL, developing personalized prevention strategies tailored to different genders and age groups. Clinicians should also enhance their awareness and diagnostic skills for AF/AFL, taking effective treatment measures promptly to address the potential increase in disease burden in the future. Consequently, implementing targeted interventions for women should be a primary focus in future healthcare strategies. Key measures include optimizing the management of postmenopausal obesity, as the decline in estrogen levels during menopause reduces anti-inflammatory effects, thereby increasing the risk of AF/AFL (44). Additionally, it is crucial to emphasize psychological interventions to address anxiety and depression (45), which are more prevalent in women and can activate the sympathetic nervous system, thereby promoting the onset of AF/AFL. These targeted approaches are essential for mitigating the heightened AF burden in women and improving overall cardiovascular health outcomes.

## Limitations

Certainly, Our investigation is bound by certain inherent limitations. Firstly, the study relies on data provided by the Global Burden of Disease (GBD 2021) database and the Global Health Data Exchange (GHDx) (1). The quality and integrity of the data probably influenced by the methodologies used for initial data collection and the standards of reporting, which may impact the accuracy of the results. This study uses population-based cross-sectional survey data, which, although large in volume and representative, may not fully reflect the individual disease progression and causal relationships. Furthermore, AF/AFL encompasses various forms, including permanent AF, long-standing persistent AF, persistent AF, paroxysmal AF, and first-diagnosed AF (5). The GBD database does not make a distinction between these conditions, thereby rendering a comparative analysis of their incidence rates infeasible. Ultimately, China is a densely populated nation comprised of numerous ethnic groups, each with its distinct living habits and conditions that vary across different regions. The GBD database does not furnish such details, and our inability to perform in-depth analyses for each region within China represents a limitation of our study.

## Conclusion

The study conducted a comprehensive analysis of the trends in the burden of AF/AFL from 1990 to 2021 globally and predicted the trend in China through 2036. While the global burden of AF/AFL is rising, China shows a mixed trend with increasing ASPR and decreasing ASMR. It was found that the burden of AF/AFL has been consistently increasing across all age groups over the past three decades. The rise is attributed to epidemiological changes, with aging being the primary contributor to mortality. The risk factors for AF/AFL are diverse, including not only traditional causes like hypertension but also psychosocial and lifestyle factors, which are particularly significant in younger individuals. There is a significant inequality in the burden of AF/AFL across countries with different levels of SDI, with medium to low SDI countries continuing to bear a heavy disease burden and having limited resources. Interestingly, China has shown a unique trend with a decline in ASMR, especially among females, suggesting improvements in management and treatment. However, the overall upward trajectory of the ASIR indicates a continuing escalation of the disease burden in the coming years. The study emphasizes the need for proactive measures to address the growing burden of AF/AFL in China and highlights the importance of gender-sensitive policies and interventions. The findings serve as a foundation for policy-making and the development of targeted intervention strategies to alleviate the burden of AF/AFL. Future research should explore the underlying mechanisms driving these trends and evaluate the effectiveness of various preventive and therapeutic approaches in different population groups.

## Data Availability

The datasets presented in this study can be found in online repositories. The names of the repository/repositories and accession number(s) can be found in the article/Supplementary Material.
